# Organoscope

**Published:** 1860-04

**Authors:** 


— THE ORGANOSCOPE is the name which has been given to a new instrument which promises to be of great service in medicine and surgery. It has been highly spoken of by M. Velpeau and other eminent Parisian authorities. Its object is to convey into the natural or pathological cavities of the human body a light, which, without burning the patient, shall facilitate exploration. For this purpose, a hollow glass tube is bent in the form of the letter Y, the two branches of which receive at their bulbous extremities the poles of a voltaic pile. The stem of the Y contains exhausted capillary tubes, which become luminous when the electric current traverses them. This stem is introduced into the cavity which is to be illuminated.

At the meeting of the Académie des Sciences, on the 22d of January, M. Fonssagrives presented a paper, of which the following is a translation:

I have long entertained the conviction that the electric light could with advantage be used in certain diagnostic examinations, and in certain surgical manipulations; for the ordinary methods of throwing light on the parts are either inadequate in intensity and in luminous radiation, or defective, in consequence of the color of their light. Besides, they are troublesome in other ways, for it is impossible to use them without hiding from view the surgeon's field of action, and it is necessary, from the great heat they give out, to hold them at a considerable distance from the surface requiring to be illuminated. Our problem, therefore, reduces itself to these three conditions: First, the luminous body required must produce little or no calorific action. Secondly, it must be capable of being condensed in tubes of minute volume and slightly varying form. Finally, the light itself must be extremely white, so as not to change the color of the organic tissues on which its rays are thrown. Thanks to the co-operation of Messieurs Du Moncel and Ruhmkorff, the problem has been satisfactorily solved. Having observed that the vacuum tubes of Geissler do not become heated under the influence of the electric light when traversed thereby, and knowing, moreover, that this light itself is more brilliant in proportion as the tubes connecting the terminal bulbs of the apparatus have a more minute diameter, M. Du Moncel thought that by taking an apparatus of this kind, in which a long tube, almost capillary, was bent upon itself and twisted like electro-magnetic multipliers, he could obtain not only a kind of luminous cylinder capable of being introduced into very narrow cavities, but also a sort of electric lantern, in certain parts of which light might be concentrated, without danger of producing heat or trouble. The first two conditions of the problem were thus satisfied. As for the color of the light in these tubes, it is well known to depend altogether upon the nature of the gas on which the vacuum was produced—being white, with certain mixed gases, such as carbureted hydrogen, carbonic acid, muriatic acid, etc. The last condition of the problem, therefore, is satisfied, if the tubes be prepared with the right kind of gas. M. Ruhmkorff, to whom the making of the tubes was intrusted, has attained a very satisfactory result; and experience has demonstrated the fact, that the light supplied by this apparatus is more than sufficient for the requirements of medicine and surgery.

Without wishing at present to trace out the precise applications to which this new method of illuminating organic surfaces is applicable, we may, however, indicate the following: First, it is a means of diagnostic exploration, and of examining the accessible cavities of the body, that their normal or pathological state may be ascertained. Secondly, it is a method of illuminating the parts during surgical operations.

The utility of this new instrument is obvious in operations in which the most serious contingent difficulties are complicated and increased by the impossibility of properly illuminating the surfaces on which the instruments are used. I may mention amongst those likely to profit in an especial manner from this new application—1, Staphylorraphy; 2, The operation for vesico-vaginal fistula, according to the American method; 3, The extirpation of uterine or naso-pharyngeal polypi; 4, The excision of the tonsils.

Moreover, certain operations in dental surgery appear likely to borrow from this mode of illumination conditions for their better and more easy performance. I may also ask whether these luminous tubes would not light up in a more complete and easy manner the field of the retina.—*Revue de Thérapeutique*, 15 *February*, pp. 103–4.

